# Coherent three-dimensional X-ray cryo-imaging

**DOI:** 10.1107/S2052252515015109

**Published:** 2015-08-20

**Authors:** Ian Robinson

**Affiliations:** a London Centre for Nanotechnology, University College, Gower St, London, WC1E 6BT, UK; b Research Complex at Harwell, Rutherford Appleton Laboratory, Didcot, OX11 0FA, UK; c Materials Science and Engineering, TongJi University, Shanghai, People’s Republic of China

**Keywords:** coherent diffractive imaging, cryo-CDI, three-dimensional imaging, three-dimensional cellular structure, coherent diffraction, X-ray imaging

## Abstract

The combination of cryogenic sample temperatures with three-dimensional coherent diffractive imaging for the case of whole frozen-hydrated cells is discussed in the light of theoretical predictions of the achievable resolution.

In this issue of **IUCrJ**, Rodriguez *et al.* (2015 ▸) from the University of California, Los Angeles, report their success in ‘Three-dimensional coherent X-ray diffractive imaging of whole frozen-hydrated cells’. This is a major achievement in the growing field of Coherent Diffractive Imaging (CDI), in which the UCLA group is one of the world leaders. The report appears to be the first time that three-dimensional CDI and cryogenic sample temperatures have both been used at the same time. In this sense, it represents the ultimate experiment.

CDI is a ‘lensless’ X-ray imaging technique, which employs a computer algorithm in place of a lens between the sample and the detector. If the detector is in the optical far-field of the sample, the diffraction pattern is a simple Fourier transform of the wavefield projected through the sample. The image is then provided by an inverse Fourier transform, subject to a ‘missing phase problem’ as in conventional crystallography. In CDI, the missing phase of this Fourier transform is synthesized by the algorithm using the fact that the diffraction pattern of a compact object can be oversampled with respect to its spatial Nyquist frequency, as first pointed out by Sayre (1952 ▸). Three-dimensional images are then obtained from the projections by conventional tomography, modified by Rodriguez *et al.* (2015 ▸) as ‘equal slope tomography’ to handle the case of a thin slab-shaped sample with limited accessibility.

CDI is maximally dose efficient because, in principle, every photon scattered by the sample can be collected and counted, without any losses associated with lenses. For three-dimensional imaging, this is particularly important because multiple views of the sample are required which can potentially increase the dose received by the sample. For biological samples, radiation dose management is desirable because dose is the main limitation to the image resolution that can be achieved. As in macromolecular crystallography, frozen samples can be used to reduce the damage rate by preventing the diffusion of free radicals. To combine this with three-dimensional imaging, where the frozen sample has to be rotated through the X-ray beam, is the important technical accomplishment of Rodriguez *et al.* (2015 ▸).

The question of the ‘ultimate’, dose-limited, resolution of an idealized CDI experiment has been discussed in two important papers in the literature: Shen *et al.* (2004 ▸) estimated the limit to be 3–5 nm, while Howells *et al.* (2009 ▸) estimated 10 nm was the resolution limit. While their detailed theoretical arguments are slightly different, both Shen *et al.* (2004 ▸) and Howells *et al.* (2009 ▸) estimate the dose tolerated by a biological sample under the optimal resolution conditions to be 10^9^ Gy. 1 Grey (Gy) corresponds to 1 J of absorbed energy per kg of sample. Rodriguez *et al.* (2015 ▸) estimate their sample received 4.55 × 10^8^ Gy and showed no sign of loss of information in the diffraction patterns. Where the experiment falls short of the ideal theoretical situation is that the resolution was only 74–99 nm, rather than in the 10 nm range. However, their experimental result is broadly consistent with the results of other groups attempting synchrotron X-ray imaging of whole cells in two and three dimensions, with and without cryo-freezing.

So maybe the theoretical models are overly optimistic for the case of biological cells? Howells *et al.* (2009 ▸) made use of the ‘dose fractionation theorem’ of Hegerl and Hoppe (1976 ▸) to argue that three-dimensional images need no more dose to achieve the same resolution as a two-dimensional projection of the same sample. However, Hegerl and Hoppe’s (1976 ▸) paper refers to a particular limit of electron microscopy of weak objects dominated by flat background due to the electron beam: ‘Such images show low contrast in comparison with the background caused by the strong primary beam. Therefore the noise determined mainly by the primary beam is independent of the signal and furthermore stationary because of the constant level of the background.’ (Hegerl & Hoppe, 1976 ▸).

In fact CDI usually operates in a different limit, collecting the diffraction pattern, well separated from the direct beam, which is blocked by a beamstop. If the experiment is designed and performed well, as the subject work of Rodriguez *et al.* (2015 ▸) appears to be, the background is negligible. This can be seen directly in the *visibility* of the diffraction fringes in the raw data: following the standard definition from optics, visibility is the difference between the maximum and minimum intensity values of a diffraction pattern, normalized to the average intensity. Fringe visibility is a useful measure of data quality because it estimates not only the amount of coherence of the incident beam, usually quite high in CDI with third-generation synchrotron sources, but also the contributions from background levels and from dose-dependent sample motions and other experimental shortcomings (Thibault & Menzel, 2013 ▸). The high visibility of the data of Rodriguez *et al.* (2015 ▸) shows that there are no serious sample stability issues and that their data are in the low-background photon-counting limit.

The theory paper of Shen *et al.* (2004 ▸) makes a rather unusual assumption about the structure of biological matter, that it is composed of randomly distributed spherical lumps with a size equal to the image resolution (of 10 nm). This leads to a rather optimistic intensity *vs* momentum transfer relationship, *I*(*Q*) ~ *Q* ^−3^. A better description would have been to assume smaller lumps of matter the size of protein molecules, macromolecular assemblies, ribosomes or nucleosomes. To the extent that these obey the assumptions of Porod’s law (smooth surfaces), we would expect *I*(*Q*) ~ *Q* ^−4^. Coherent diffraction from such a random distribution of ideal spheres would give the spherical form factor modulated by speckles with the average intensity per speckle falling as *I*(*Q*) ~ *Q* ^−4^. When the intensity per speckle drops to the minimum significant level, often chosen to be 25 photons to meet the Rose criterion of a signal/noise ratio of 5, we reach the resolution limit. However, for three-dimensional CDI, the required number of tomographic slices increases linearly with *Q* as well, in order that every speckle be oversampled in the rotation direction. So we end up with the expectation for three-dimensional CDI that the dose should scale with *Q*_max_^5^, the resolution to the fifth power. This may explain why the experimental resolution found by Rodriguez *et al.* (2015 ▸) is so much lower than had been anticipated.
